# Inverting the Wollaston Illusion: Gaze Direction Attracts Perceived Head Orientation

**DOI:** 10.1177/20416695211046975

**Published:** 2021-10-20

**Authors:** Heiko Hecht, Ariane Wilhelm, Christoph von Castell

**Affiliations:** Department of Psychology, 9182 Johannes Gutenberg-Universität Mainz, Mainz, Germany

**Keywords:** visual perception, gaze direction, facial features, Wollaston illusion

## Abstract

In the early 19th century, William H. Wollaston impressed the Royal Society of London with engravings of portraits. He manipulated facial features, such as the nose, and thereby dramatically changed the perceived gaze direction, although the eye region with iris and eye socket had remained unaltered. This Wollaston illusion can be thought of as head orientation attracting perceived gaze direction when the eye region is unchanged. In naturalistic viewing, the eye region changes with head orientation and typically produces a repulsion effect. Here we explore if there is a flip side to the illusion. Does the gaze direction also alter the perceived direction of the head? We used copies of the original drawings and a computer-rendered avatar as stimuli. Gaze direction does indeed alter perceived head orientation. Perceived head orientation is biased toward the direction of gaze.

## Introduction

When judging the gaze direction of a person we face, head orientation is of utmost importance. Features of the head, such as a prominent nose, specify head orientation. Given an unchanged eye region, head orientation attracts perceived gaze direction. This has been famously demonstrated by Wollaston at the Royal Society almost 200 years ago (see Hecht et al., 2020; Wollaston, 1824). The story is rather different when the eye region does not remain unchanged. Perceived gaze is changed as a function of eye-eccentricity (Todorović, 2009). And as a person's head is, say, turned to an observer's left while gaze is maintained on the observer, iris and pupil become more eccentric with respect to the eye socket and the rest of the head. This results in the observer's impression that the head repulses gaze direction. In other words, an unchanged eye region causes perceived gaze to be attracted by head orientation, whereas an eye region that changes eccentricity in a naturalistic manner as the head is turned, produces the opposite effect of repulsion. Gaze direction appears to be shifted away from the direction in which the head is oriented. This state of affairs has been termed ‘dual route’ (Otsuka et al., 2014), but it amounts to two factors of opposite sign influencing perceived gaze, namely, the repulsing eccentricity effect and the attracting Wollaston effect (see also Balsdon & Clifford, 2017).

In the current paper, we investigate the other side of the coin. If features that indicate an altered head orientation exert an effect on perceived gaze direction, is the opposite likewise the case? Does an altered gaze direction exert an effect on perceived head orientation? We hypothesized that this would be the case. Unfortunately, one cannot keep the eye region unchanged and change gaze direction at the same time. Thus, the Wollaston experiment cannot be perfectly inverted. However, it is possible to vary gaze direction and head orientation independently. And this is what we did. We also added a set of more controlled stimuli.

Note that the Wollaston drawings do not reveal where the depicted head was looking. As a matter of fact, even a photograph of an actual person looking at an object in a well-defined place cannot be reverse-engineered to reveal the exact head orientation and/or gaze direction without information about the three-dimensional (3D) shape of the eyeball, the eye socket, etc. In contrast, if the 3D shape of a face is known, we can determine exactly how a 2D picture of this face should look provided its gaze is directed at a given point in space, say at an object 1 m away and 10° to the side. Consequently, we created an additional set of stimuli on the basis of a virtual head, which we constructed with a 3D rendering tool (‘make human’). This virtual head could be rotated around its vertical axis. Into this head, we placed a pair of eyes that could rotate independently of the head. This gave us a basis for a systematic and independent variation of 3D head orientation and gaze direction.

Since it had to this date been unknown to what extent the perceived head orientations change within Wollaston's drawings (with the exception of a qualitative result that they do; Todorović, 2006), we decided to do two things. First, we revisited the original Wollaston drawings where the eye region remains unaltered and had observers judge perceived head orientation. This complements the existing gaze direction data we have on Wollaston's drawings, where perceived gaze was attracted by the drawn facial features to an impressive extent of up to 15° (Hecht et al., 2020). Would the eyes exert an effect of similar magnitude in the original drawings, or had these drawings been optimized to demonstrate the gaze-altering power of facial features?

We also asked for perceived gaze direction (similar to Hecht et al., 2020) to have comparable data for perceived head orientation and perceived gaze direction on the same set of observers. Here, we should replicate the attraction effect of head orientation on judged gaze direction. We should also learn about the extent of head orientation changes based on the reduced features transported by the drawings.

Second, for a given gaze direction of the computer-generated avatar, we produced stimuli with head orientations corresponding to or deviating to the left and right from the given gaze direction. For all of these stimuli, subjects had to judge perceived gaze direction and perceived head orientation in separate blocks. Thus, the current study compared judgments of both head orientation and gaze direction across drawings and 3D-based pictures on the same group of observers.

It is not easy to make predictions about whether gaze direction should attract or repulse perceived head orientation. One could argue that in social contexts, we need to know where the other person is looking and could not care less about head orientation. However, since we are already unable to ignore the irrelevant head orientation when asked to judge gaze, we likely factor in gaze when asked to judge head orientation. We predicted that gaze direction is so prominent that it biases perceived head direction toward the direction of the gaze.

## Method

### Design

**Figure 1.**
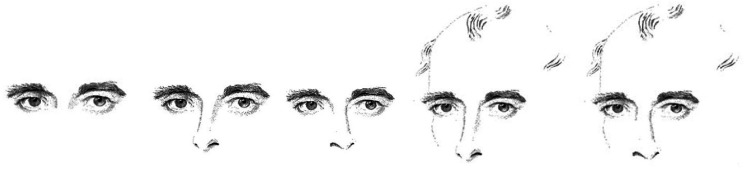
Selection of the original heads published by Wollaston (1824). From left to right, the heads correspond to the stimuli 3, 4, 5, 9, and 11 used by Hecht et al. (2020). Note that the eye-region is identical in all cases.

As judged head orientation had not previously been recorded, we prefaced the experiment with an assessment of the original Wollaston stimuli. We chose five drawings from the original set (corresponding to stimuli 3, 4, 5, 9, and 11 from Hecht et al., 2020) and had subjects judge the perceived gaze direction and head orientation of the person in two separate blocks. The drawings are depicted in Figure 1. Each drawing was presented twice for each task.

Next, we presented all virtual head stimuli. The virtual head assumed five gaze angles that maintained observer eye-height but deviated horizontally from mutual gaze to the left or to the right by −10°, −5°, 0°, 5°, 10°. Then, for each gaze direction, five head orientations were created, deviating by −10°, −5°, 0°, 5°, 10° from the gaze. We crossed gaze direction and head orientation fully, resulting in 25 unique stimuli. They were presented at different random orders, twice with the task to judge gaze direction, and twice with the task to judge head orientation. The order of whether subjects started with the gaze judgment task or with the head orientation task was counterbalanced, such that half of the subjects started with each task. Thus, subjects judged a total of 5 (gaze direction −10°, −5°, 0°, 5°, 10°) by 5 (head orientation relative to gaze −10°, −5°, 0°, 5°, 10°) by 2 (repetition) by 2 (judgment) stimuli (summing to 100 trials).

### Subjects

Thirty-eight subjects (6 male, 31 female, and 1 diverse) volunteered to participate in the experiment. They were recruited using university mailing lists and gave informed consent in accordance with the Declaration of Helsinki. Because only harmless visual stimuli were presented, no physiological parameters were measured, and no wrong or misleading information was given to the subjects, according to the guidelines of the local ethics board (Department of Psychology in Mainz), no vote of the ethics board was necessary. During debriefing, all subjects reported to have been unfamiliar with the Wollaston drawings prior to the experiment. Their average age was 23.74 years (*SD* *=* 4.12 years). Two subjects chose not to judge the original Wollaston heads, and two subjects produced too many missing data and were removed from the analysis of the virtual head data. With these exceptions, 36 data sets were obtained with the Wollaston stimuli and 36 data sets with virtual heads. Of the latter, 18 subjects judged gaze direction first and the other 18 judged head orientation first.

**Figure 2.**
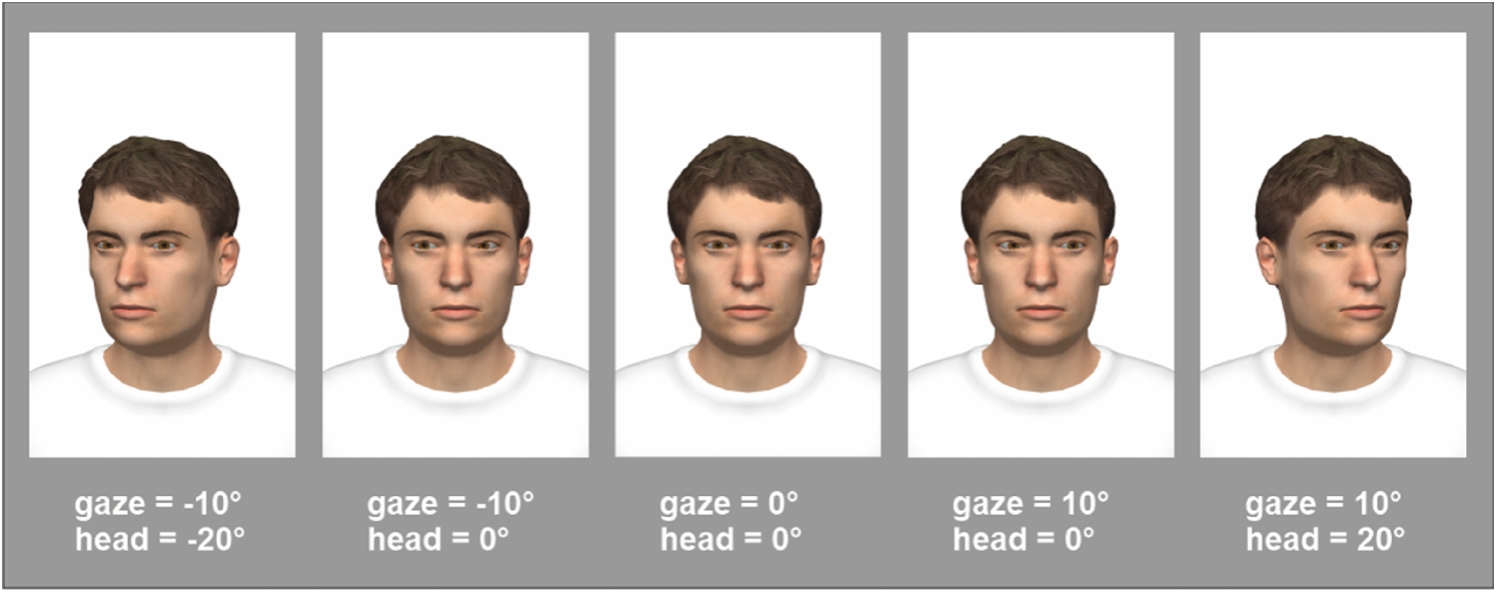
Examples of the avatar stimuli constructed using a 3D renderer. Gaze angles and head orientations are indicated in degrees. 0° is straight ahead for the subject, negative values indicate gaze/head orientation to the subject's left. The entire set of stimuli can be viewed online at https://osf.io/wn34x/?view_o.

Stimuli were created using the virtual environment tool ‘make human’ and rendered using the software Vizard 5. The head including eye socket was rendered in 3D, as were the eyes. For each stimulus, a particular head orientation and gaze angle were set, such that the eyes converged at the observer's bridge of the nose, for the case of direct gaze. The convergence point was at 100 cm from the avatar, at the physical position of the subject. For averted gaze conditions, the avatar's eyes converged on a point on the horopter. Then, a screenshot was produced for each stimulus condition. Four example stimuli of the virtual head are shown in Figure 2.

The screenshots could thus easily be presented at different random orders. Note that the eyelids and eye sockets, as well as all other facial features and expressions, remained constant for all combinations of head orientation and gaze angle. That is, the eyeball was rotated behind the eye socket. Potential minute stretching of the skin due to eye rotation was not modeled because the make-human models, as all such models, had a finite number of parameters available that simulated bone and muscles. Skin elasticity would have exceeded the limits of the model and might have interacted with perceived gaze direction if done crudely.

### Apparatus

**Figure 3.**
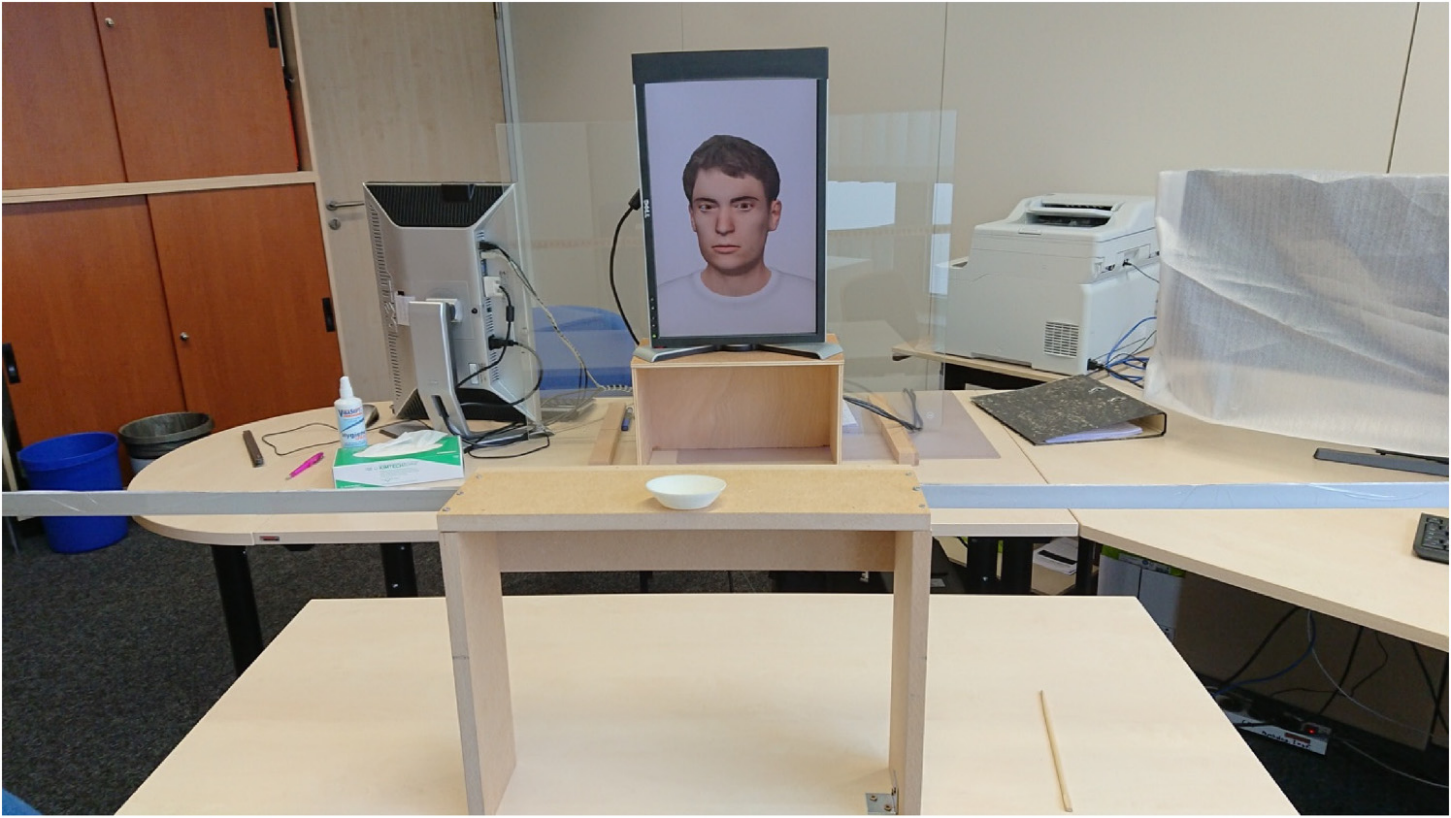
Setup of the experiment. Note the chin rest for the subject. The horizontal bar underneath it had cm unit values painted on the other side, only visible to the experimenter, who sat behind the display monitor, such that his/her face was occluded to the subject.

The stimuli were presented on a 22” display with a resolution of 1050 × 1680 pixels (horizontal × vertical). The top two centimeters of the display were covered with black tape as they displayed running stimulus numbers necessary for the experimenter to identify each stimulus on the separate display, which mirrored the stimulus but was only visible to the experimenter. During the experiment, subjects sat on a height-adjustable chair with their eye position horizontally centered to the monitor and vertically level with the stimulus head. The stimulus head subtended a vertical visual angle of 13.4° from chin to top. Attached to the apparatus holding the chin rest was a horizontal bar with cm unit values, which were visible only to the experimenter sitting behind the monitor facing away from the subject, which mirrored the display of the first monitor (see Figure 3).

### Procedure

First, we presented all of the five original Wollaston drawings once for a gaze direction judgment, and once for a head orientation judgment. Half of the subjects judged gaze direction first, the others started with the head orientation task. Next, we presented the avatars. Subjects alternatingly started either with the gaze judgment or the head orientation task and completed all trials of this dependent variable. After a short break, all stimuli were presented again in different random orders and the other judgment task had to be performed. Subjects indicated perceived gaze direction (and perceived head orientation in the other block) by pointing to the location on the horizontal bar where the avatar's gaze (or head direction) would intersect the plane of the bar. The experimenter then recorded the value in cm. Later this distance value from straight-ahead was converted to degrees. After the experiments, we asked all subjects, whether they had been familiar with the Wollaston drawings prior to the experiment or whether they had previously participated in experiments on gaze perception. This was not the case.

## Results

### Wollaston's Original Heads

**Figure 4.**
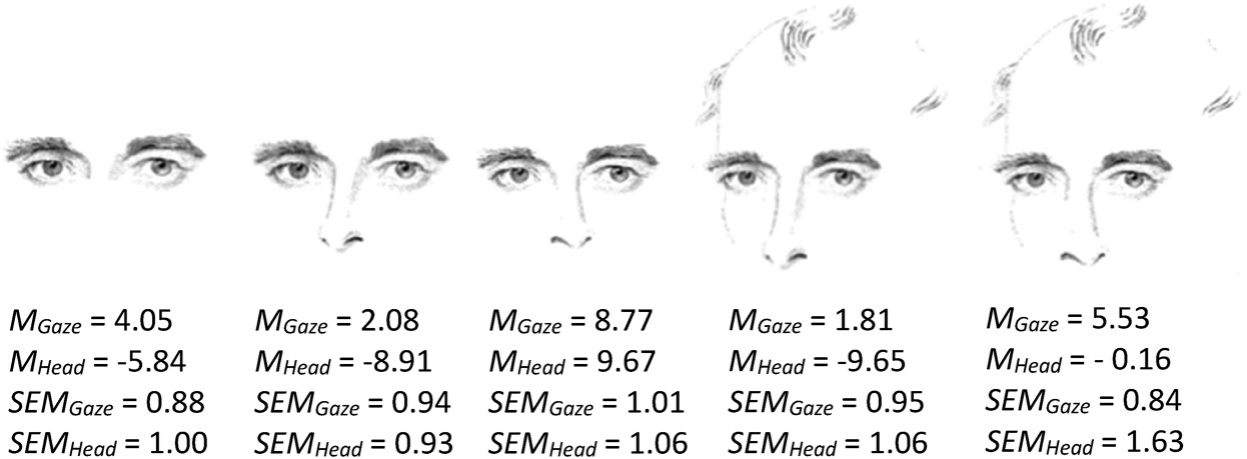
Mean judged gaze direction and judged head orientation in degrees. Negative values indicate directions to the subject's left. The lower rows indicate the matching standard errors of the mean for gaze direction and head orientation.

Figure 4 depicts the mean judged gaze directions and head orientations averaged across all subjects and both stimulus repetitions. The eye region by itself produced the impression of the gaze being directed slightly to the subject's right. The head, in contrast, was judged to be turned somewhat to the left. The subjects presumably based their judgment of head orientation on the slight shadow asymmetry of the bridge of the nose and the slight eccentricity of the iris. When inspecting the values for the second and third stimulus merely differing by the shape of the nose, it is obvious that the direction of the nose attracts the gaze direction by less than it attracts head orientation (difference of means for gaze = 6.7°, *t*(35) = −6.72, *p* < .001, Cohen’s (1988) d*_z_* = 1.12; difference of means for head orientation = 18.6°, *t*(35) = −11.48, *p* < .001), d*_z_* = 1.97. This appears reasonable as the nose is attached rather firmly to the head.

### Artificial Avatar Heads

**Figure 5.**
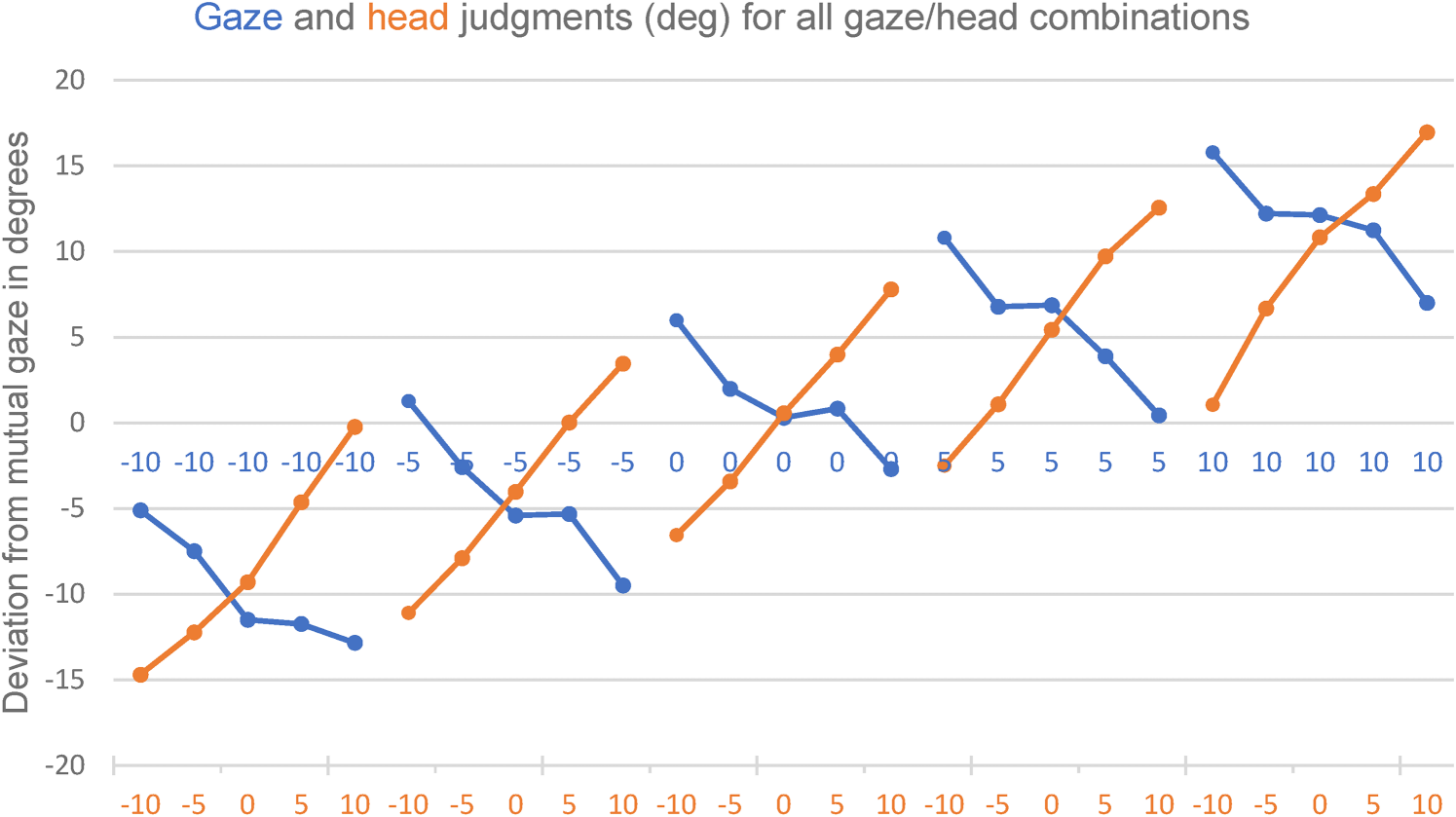
Mean judged gaze directions (blue values) and judged head orientations (orange values). Note that head orientation was varied relative to gaze by 0, 5, and 10 to each side. Thus, blue numbers indicate true gaze direction, orange numbers indicate head orientation relative to gaze. Negative values indicate directions to the subject's left. All values in degrees.

With regard to the virtual head, 4 missing values were replaced. One missing gaze angle was substituted with the corresponding value from the repetition of the same stimulus. Three such substitutions were made for missing head orientation values. The averaged mean values for each stimulus are displayed in Figure 5.

**Figure 6.**
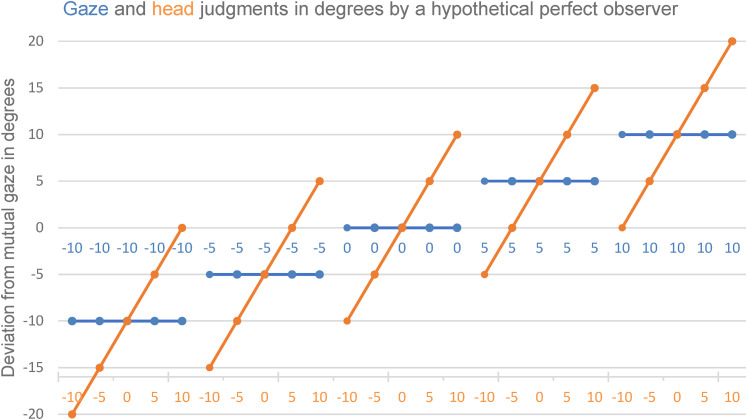
Saw-tooth pattern of perceived gaze direction (blue) and head orientation (orange) as would be expected from an ideal observer immune to any bias. For each of the five gaze directions, head orientation was aligned with the gaze or rotated by 5° and 10° to the left or right.

Note that we had varied head orientation relative to the given gaze directions ranging from −10° to 10°. Accordingly, the expected results for an ideal observer who is unaffected by impressions of eccentricity or head orientation should produce a saw-tooth pattern as illustrated in Figure 6. The subjective judgments should be compared to such an ideal observer.

With respect to the gaze judgments, we see a very clear pattern. Perceived gaze direction is repulsed by head orientation. Take for instance the first five data points in Figure 5. For the gaze directed 10° to the left (as seen by the subject), judged deflection of gaze relative to the observer is underestimated when the head is turned even more to the left, and it is overestimated when the head is turned to the right. Gaze is perceived less centered with respect to the head than it actually is.

An effect of gaze direction on perceived head orientation is less obvious. The former appears to attract perceived head orientation in particular if the head is turned farther away from straight-ahead than is the gaze.

We calculated two separate multiple linear regressions for each of the 36 subjects to predict their individual estimates for gaze direction and head orientation from the experimental manipulations of actual gaze direction and head orientation. Both predictors were entered simultaneously into the model. We report unstandardized beta-coefficients so that the weights directly indicate the perceived change in degrees in response to a one-degree change in the respective predictor. Averaged across subjects, we found that the estimates could be best predicted by the equations:

$$\begin{array}{rl} \mathrm{gaze}\_\mathrm{directio}n_{\mathrm{perceived}} & =.94[.46;1.41]\\ & \;+1.06[.90;1.22]\times \mathrm{gaze}\_\mathrm{direction}\\ & \;-0.42[-.52;-.31]\times \mathrm{head}\_\mathrm{orientation}, \end{array}$$

$$\;R^{2}=.82[.78;.87],\mathrm{and}$$

$$\;\mathrm{head}\_\mathrm{orientatio}n_{\mathrm{perceived}}=.68[.30;1.06]$$

$$\begin{array}{rl} & \;\;\;\;\;\;\;+.90[.79;1.02]\times \mathrm{gaze}\_\mathrm{direction}\\ & \;\;\;\;\;\;\;+.75[.63;.87]\times \mathrm{head}\_\mathrm{orientation}, \end{array}$$

$$\;R^{2}=.93[.92;.95].$$

Perceived gaze direction reflects true gaze direction and a repulsing effect of head orientation. Perceived head orientation was predicted more strongly by gaze direction than by head orientation, as confirmed by a paired-samples *t*-test (two-tailed) comparing the individual beta-coefficients for gaze direction and head orientation, *t*(35) = 3.76, *p* = .001, *d_z_* = .63. Considering our design, where head orientation was defined relative to gaze direction, this means that an equidirectional rotation of head and gaze led to a stronger change in perceived head orientation than a change in head orientation alone. Note that the latter led to a deflection of head orientation from gaze direction. Thus, perceived head orientation reflects the true head direction and an attracting effect of gaze. The 95% confidence intervals of the beta-weights (the values in brackets indicate the lower and upper bound, respectively) additionally indicate high levels of between-subject agreement in the weighting of head and gaze information when judging gaze direction and head orientation.

To gain further insight into the effects of head orientation and gaze direction on the gaze and head judgments, we calculated the estimation error for gaze direction and head orientation separately for each subject and combination of head orientation and gaze direction, that is the absolute deviation in degrees between perceived and actual gaze direction or between perceived and actual head orientation. Deviations to the left from the perspective of the subject were coded as negative differences, deviations to the right were given positive values. We calculated one repeated measures analysis of variance (rmANOVA) for the estimation error for gaze direction, and another for the estimation error for head orientation, using a univariate approach with Huynh and Feldt (1976) correction for the degrees of freedom (correction factor $\tilde{\varepsilon }$ ). The within-subject factors were head orientation and gaze direction.

**Figure 7.**
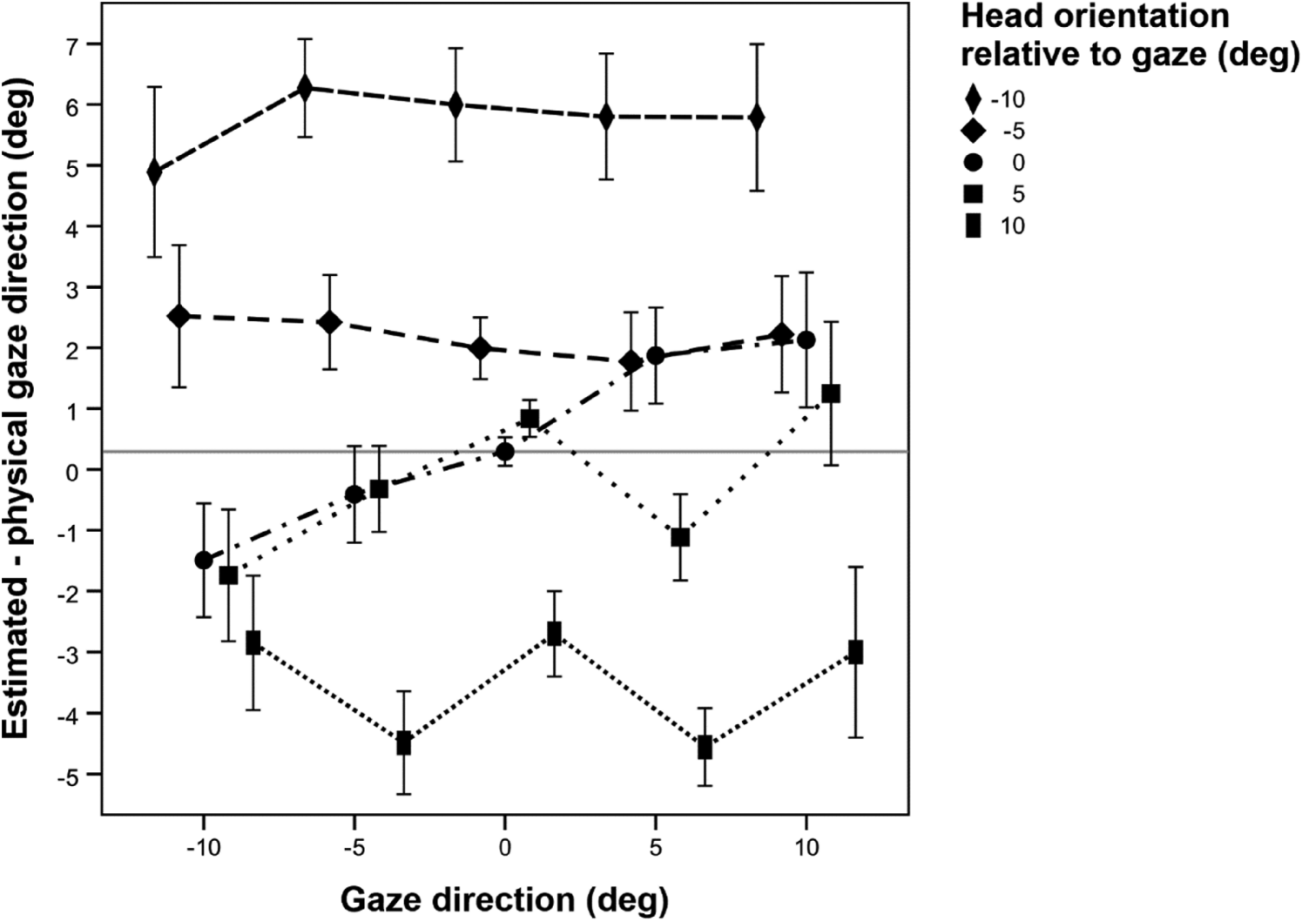
Mean estimation error for gaze direction as a function of gaze and head direction. Negative values indicate deviations to the subject's left. The gray solid line shows the baseline when both gaze and head of the avatar pointed directly at the observer. All values in degrees. Error bars show ±1 standard error of the mean (SEM) of the 36 individual estimates in each condition.

We first consider the *estimation error for gaze direction*. Figure 7 shows the deviation of perceived gaze from actual gaze as a function of head orientation and gaze direction. In the rmANOVA, the effect of head orientation was significant, *F*(4, 140) = 50.26, *p* < .001, $\eta_{p}^{2}$ = .59, $\tilde{\varepsilon }$ = .41. Averaged across the five directions of gaze, as the head rotates from left to right relative to gaze, the estimation error for gaze changes from 5.75° (*SD* = 4.45°) to the right (for the leftward looking head) to 3.52° (*SD* = 3.18°) to the left (for the rightward pointing head). Thus, when the head is directed to the side, it does repel the gaze direction. It does so in all cases, as the effect of gaze direction was not significant, *F*(4, 140) = .66, *p* = .623, $\eta_{p}^{2}$ = .02, $\tilde{\varepsilon }$ = .33. However, there was a small but significant head orientation × gaze direction interaction, *F*(16, 560) = 2.26, *p* = .008, $\eta_{p}^{2}$ = .06, $\tilde{\varepsilon }$ = .78. In particular, when the avatar's head was aligned with gaze, the estimation error for gaze varied somewhat as a function of gaze direction. With the rotation of gaze from left to right, the mean estimation error changed gradually from 1.49° (*SD* = 5.61°) to the left to 2.12° (*SD* = 6.65°) to the right, which means that, on average, subjects slightly overestimated gaze eccentricity when the avatar's head was aligned with gaze. Thus, gaze was perceived rather accurately when the head pointed to the subject.

Across all experimental conditions, the mean estimation error for gaze direction was +.94° (*SD* = 1.41°), which indicates a slight shift of the perceived gaze direction to the right from the observer's perspective. We hold slight asymmetries in the head responsible for this bias. Note that we opted against a perfectly symmetric head, it looked less natural than the current version.

**Figure 8.**
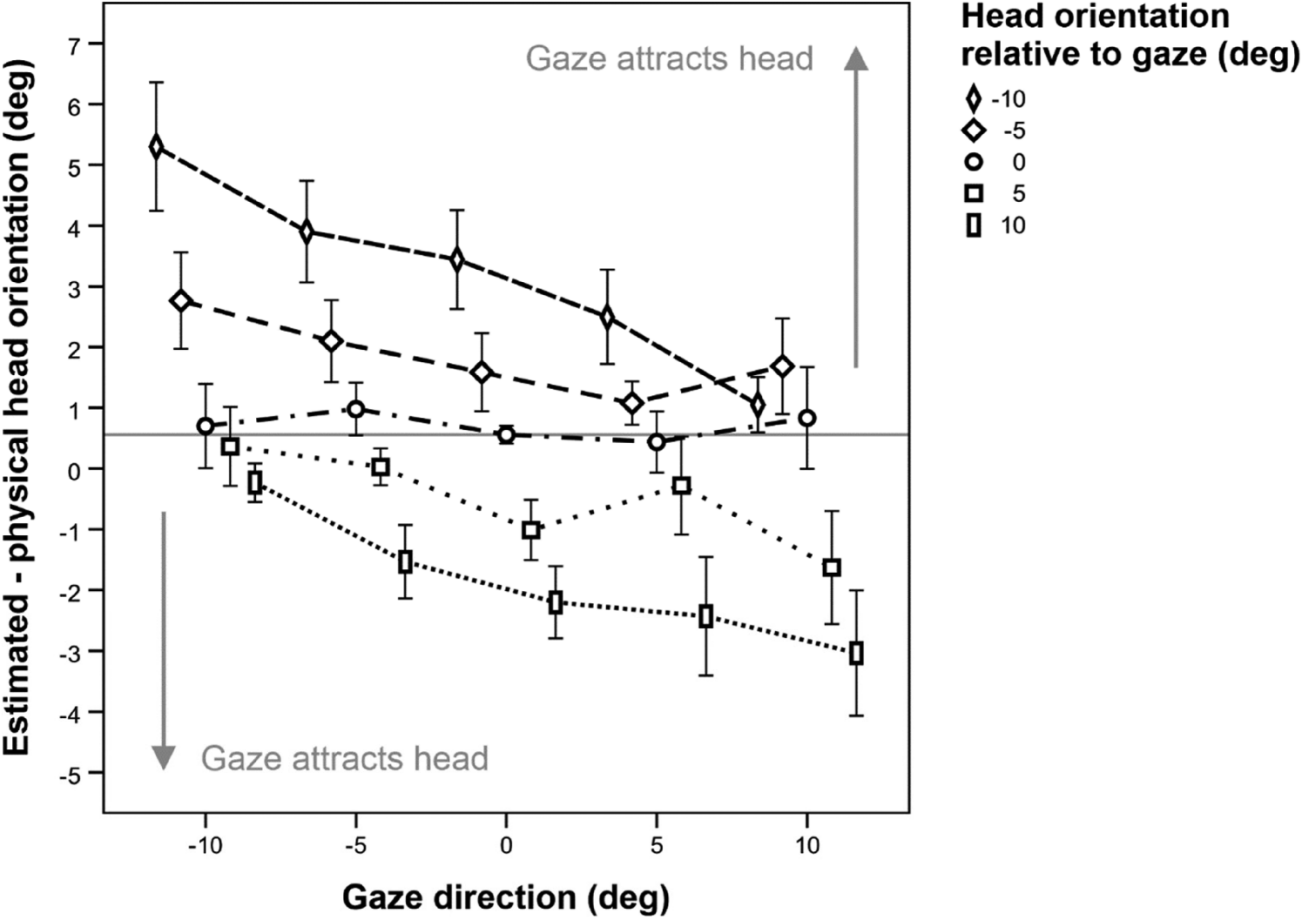
Mean estimation error for head orientation as a function of gaze and head direction. Negative values indicate deviations to the subject's left. For a deflection of the head to the left relative to gaze (dashed lines), a positive estimation error indicates an attraction of the head by gaze. Conversely, when the head was deflected to the right relative to the gaze (dotted lines), a negative estimation error indicates that the gaze attracted the head. The gray solid line shows the mean deviation from zero when both gaze and head of the avatar pointed directly at the observer. All values in degrees. Error bars show ±1 SEM of the 36 individual estimates in each condition.

We next consider the *estimation error for head orientation*, see Figure 8. Across all experimental conditions, the mean estimation error for head orientation was merely +.68° (*SD* = 1.13°) and thus showed a slight rightward shift, as did the estimation error for gaze direction (see above). We suspect that the deviation from 0 is owed to a slight natural asymmetry in the head.

Averaged across the variations of head orientation, the estimation error for head orientation was 1.78° to the right for gaze directed maximally to the left and changed to .22° to the left when gaze directed maximally to the right. In the rmANOVA, the effect of gaze direction missed significance, *F*(4, 140) = 2.85, *p* = .093, $\eta_{p}^{2}$ = .08, $\tilde{\varepsilon }$ = .30, accompanied by a significant head orientation × gaze direction interaction, *F*(16, 560) = 22.36, *p* = .001, $\eta_{p}^{2}$ = .08, $\tilde{\varepsilon }$ = .70. The effect of head orientation was likewise significant, *F*(4, 140) = 16.76, *p* < .001, $\eta_{p}^{2}$ = .32, $\tilde{\varepsilon }$ = .29. As can be seen in Figure 8, the estimation error for head orientation varied systematically as a function of head orientation. Averaged across gaze, the mean estimation error changed gradually from 3.24° to the right for the maximum deflection of the head to the left to 1.89° to the left for the maximum deflection of the head to the right, which indicates a slight overall underestimation of the avatar's head orientation.

When looking more closely at Figure 8, we see that the effect of gaze direction on the estimation error for head orientation varies as a function of head deflection relative to gaze. The effect is most pronounced when the head is maximally deflected from gaze, and practically zero when head orientation coincides with a gaze. Eye-eccentricity might be responsible for this effect (see Discussion). Head orientations that deviate strongly from gaze (−10° and +10° deflection relative to gaze) were clearly attracted by gaze, most prominently when the head was more averted from the observer than the gaze, while moderately deviating head orientations (−5° and +5° deflection relative to gaze) were less attracted by gaze. Perceived head orientation was by and largely unaffected by gaze direction when the head was aligned with a gaze.

In sum, our results show a consistent repulsion effect of head orientation on perceived gaze direction. The effect of gaze direction on perceived head orientation turned out to be less universal. Only when gaze direction clearly differed from head orientation, that is head orientation was deflected 10° to the left or to the right relative to gaze direction, did our results show a pronounced influence of gaze direction on perceived head orientation. In this case, gaze direction attracted perceived head orientation, contrary to the repulsion of perceived gaze direction by head orientation.

## Discussion

In the present study, we have examined the interaction of perception of gaze direction and head orientation. Whereas the influence of head features on gaze direction had been famously well researched, the opposite influence of gaze direction on perceived head orientation had not previously been investigated. We did so with two entirely different types of stimuli. Using some of the original Wollaston drawings (Hecht et al., 2020), we have first replicated the classical Wollaston effect, namely that the orientation of further facial features attracts perceived gaze direction, although the eye region remains unchanged. In the following main study, we used an avatar head by means of which we systematically manipulated gaze direction and head orientation within the approximate range of typical natural viewing situations. Here, we found that the head orientation did not attract perceived gaze, but instead substantially repelled it. Conversely, we found an attraction effect, albeit weaker, of gaze direction on head orientation. How can these findings be brought together?

With regard to the avatar head, there are two fundamentally different ways to think about how head orientation and gaze direction combine to create the impression of direct or averted gaze. One the one hand, one can start with the 3D world. There, a given head has a well-defined orientation with respect to an observation point. The direction in which the nose points – assuming it is well formed and well attached to the head – specifies head orientation. We call the orientation when the nose is normal to the observation point as a head orientation of 0°. A similar case can be made for gaze direction. When the surface normal of the eyeball points to the observer (or more precisely, when the pupillary axes from both eyes intersect on the observer's bridge of the nose), we have called it gaze direction 0°. Thus, an ideal observer in the 3D world should judge a person to look at her whenever gaze direction is 0°. Head direction should be irrelevant. Any deviation from 0°, as induced by context cues or otherwise, can accordingly be defined as error or bias. This is pretty much what we have done thus far. In analogy, this holds for all other gaze directions involving averted gaze. We find that perceived gaze is rather accurate as long as the head is aligned with a gaze. If, however, the head is turned relative to the gaze (rotated around the yaw-axis), it appears to repel perceived gaze direction. A bias arises such that perceived gaze is judged to be deflected to the left when the head is turned to the right – and vice versa.

On the other hand, one could look at things from a constructive pictorial perspective, such as an artist would assume when painting a portrait that is meant to look, say, directly at the observer. Here, the real gaze direction and head orientation are unknown. Bias or error in absolute terms are devoid of meaning. Nonetheless, the cues that determine perceived gaze and head direction in a portrait can be well-described, as has been done by Todorović (2006). Eccentricity of the main facial features (eyes, mouth, and nose) relative to the contour of the head determines impressions of head orientation, and eccentricity of the iris and pupil within the eye socket determines perceived gaze direction relative to the head. Note that they interact. Thus, depending on whether or not a picture has a 3D origin, one is confined to eccentricity values or one can use the corresponding directional values as pertaining to the 3D origin. In the cases where we used the avatar, the original 3D data about head orientation and gaze direction are known, and we take the 3D view to be more convenient and more powerful. Note also that the experiments of West (2013, 2015) nicely demonstrate that observers’ gaze direction judgments are identical, regardless of whether stereoscopic 3D pictures or 2D pictures of the same stimulus are presented.

### Anisotropy Between Effects of Head on Gaze and Gaze on Head

Why is there a repulsive effect of head orientation on perceived gaze direction? Note that this is opposite to the Wollaston effect. In Wollaston's drawings, the eye socket and the eccentricity of the iris within the socket remain unchanged. Merely the perspective on the nose and, if present, other facial features change. If one eliminates all of the facial features in a turn-taking fashion, the remaining features all attract perceived gaze direction (see Figure 4). We hold that this is the case for the Wollaston stimuli because iris eccentricity does not change in these 2D stimuli. In a natural 3D stimulus, in contrast, iris eccentricity changes visibly. It is a very prominent cue; the iris shifts within the eye socket and is increasingly occluded by the eyelid as the head is turned. Note that for the latter case, iris eccentricity is tightly coupled to head orientation and gaze direction. This can be illustrated with the example of our avatar stimuli: We have measured 2D eccentricity values by calculating (in cm on the picture surface) the eccentricity of the iris with respect to the center of the eye socket, relative to the visible horizontal extent of the eye socket (again in cm of the picture surface). Iris eccentricity of the left eye and the right eye can be reliably predicted by the combination of 3D head and gaze information (entered as unstandardized predictors), iris eccentrictiy_left eye_ = −.068 + .008 × gaze direction –.011 × head orientation, *R*^2^ = .85, and iris eccentrictiy_right eye_ = .076 + .007 × gaze direction –.013 × head orientation, *R*^2^ = .99, respectively. In Wollaston's pictures, this highly systematic, and anticipated change in iris eccentricity does not happen. In other words, only the facial features change and thus attract the gaze of the unchanged eye region. In the case of the 3D head, in contrast, the change in iris eccentricity is so prominent that it not only changes perceived gaze direction but changes it more than would be appropriate. If the 3D avatar is initially gazing at the observer (head and gaze aligned straight ahead) and then the eyeball maintains gaze direction while the head is turned, perceived gaze does not remain stable. It deviates against the turn directions of the head. Apparently, iris eccentricity receives more weight than it should. We maintain that an over-emphasis of iris eccentricity is responsible for the effect that 3D head direction repulses perceived gaze direction. And it does not really matter whether the natural 3D head is presented stereoscopically or as a 2D picture.

Why is there an attracting effect of gaze direction on perceived head orientation? This question is harder to answer. Given that iris eccentricity is a powerful cue, as soon as there is a dissociation of gaze direction and head orientation, iris eccentricity will arise. Take an avatar which starts to turn its head to the left while its eyes continue to fixate the observer. Iris eccentricity increases the farther the head turns. In a 0°-head, such eccentricity would indicate a gaze shift to the right. A pull of the perceived head orientation toward the actual or perceived gaze direction would be plausible if the eyes receive particular attention. We suppose that this is the case. Thus, the eyes being of prime importance are treated differently in the situation where gaze has to be judged compared to the situation where a head orientation has to be judged. Also, the prototypical gazing head may well be one where head orientation and gaze direction are aligned, which could produce a bias toward alignment. And if the gaze is of prime importance, such bias toward the canonical alignment of head and gaze would then adjust perceived head orientation^1^. When judging gaze, iris eccentricity is suggestive of off-center gaze. When judging head orientation, eccentricity should be irrelevant, but the gaze is apparently so salient that it exerts some attraction on perceived head orientation. Note that the effect of iris eccentricity (due to head deflection relative to gaze) on perceived gaze produces larger errors than the effect of gaze direction on perceived head orientation (compare Figures 7 and 8).

### Limits of the Simulation

Our stimulus avatar was programmed such that its eyes always converged on a point of the observer's bridge of the nose between the eyes, or that its eyes fixated a point on the avatar's horopter when its gaze was averted from the observer. That is, the axes of the avatar's eyes converged 1 m in front of the avatar. Now, depending on which eye of the avatar the observer focuses, perceived gaze direction might differ slightly. This usually goes unnoticed and is of course the case when we engage in mutual gaze with a real person at such close distance. When attending to the eyes at a close distance, we notice that the observer is cross-eyed, as was the case in the study by West (2015) where a looker's eyes converged at 0.8 m. This also explains why the range of gaze angles considered as mutual is quite generous, about 10°, and why this range narrows to a little more than half this value when one eye is covered by an eye patch (see Gamer and Hecht, 2007). Moreover, although the eyelids and the eyeballs were modeled in detail and independently for our avatars, the skin was not attached as perfectly to the eyeball as typical for real-world eyes. This might have introduced some additional variability in the gaze estimates, and it could have produced the slight baseline shift that emerged. However, none of our subjects mentioned that the avatar looked strange or that it had been difficult to determine gaze direction or head orientation.

## Conclusions

We conducted an experiment to further probe into the Wollaston effect. To do so, we created 2D pictures from 3D avatars, whose head orientation and eye direction could be varied precisely and independently. Our observers not only judged the perceived gaze direction of the avatar, as typically done, but they also judged perceived head orientation. We found two errors of opposite signs. First, the orientation of the head repulsed perceived gaze direction. This is a strong effect, which we blame on the salience of iris eccentricity. Second, gaze direction attracted perceived head orientation. This is a comparatively weaker effect, which we blame on the primacy of gaze. It can be thought of as the inverted Wollaston effect. Just as independent manipulation of head orientation attracts perceived gaze, so does manipulation of gaze attract perceived head orientation.

## Notes

1. We thank the anonymous reviewer who pointed out this mechanism.

## References

[bibr1-20416695211046975] Balsdon T. Clifford C. W. (2017). A bias-minimising measure of the influence of head orientation on perceived gaze direction. Scientific Reports, 7, 41685. 10.1038/srep41685 28139772 PMC5282588

[bibr2-20416695211046975] Cohen J. (1988). Statistical power analysis for the behavioral sciences (2nd ed.). L. Erlbaum Associates.

[bibr3-20416695211046975] Gamer M. Hecht H. (2007). Are you looking at me? Measuring the cone of gaze. Journal of Experimental Psychology: Human Perception and Performance, 33(3), 705–715. 10.1037/0096-1523.33.3.705 17563231

[bibr4-20416695211046975] Hecht H. Siebrand S. Thönes S. (2020). Quantifying the Wollaston illusion. Perception, 49(5), 588–599. 10.1177/0301006620915421 32279602

[bibr5-20416695211046975] Huynh H. Feldt L. S. (1976). Estimation of the box correction for degrees of freedom from sample data in randomized block and split-plot designs. Journal of Educational Statistics, 1(1), 69–82. 10.3102/10769986001001069

[bibr6-20416695211046975] Otsuka Y. Mareschal I. Calder A. J. Clifford C. W. (2014). Dual-route model of the effect of head orientation on perceived gaze direction. Journal of Experimental Psychology: Human Perception and Performance, 40(4), 1425–1439. 10.1037/a0036151 24730742 PMC4120707

[bibr7-20416695211046975] Todorović D. (2006). Geometrical basis of perception of gaze direction. Vision Research, 46(21), 3549–3562. 10.1016/j.visres.2006.04.011 16904157

[bibr8-20416695211046975] Todorović D. (2009). The effect of face eccentricity on the perception of gaze direction. Perception, 38(1), 109–132. 10.1068/p5930 19323141

[bibr9-20416695211046975] West R. W. (2013). The effect of head turn and illumination on the perceived direction of gaze. Perception, 42(5), 495–507. 10.1068/p7343 23964376

[bibr10-20416695211046975] West R. W. (2015). Differences in the judged direction of gaze from heads imaged in 3-D versus 2-D. Perception, 44(7), 727–742. 10.1177/0301006615594702 26541051

[bibr11-20416695211046975] Wollaston W. H. (1824). On the apparent direction of eyes in a portrait. Philosophical Transactions of the Royal Society of London, 114, 247–256. 10.1098/rstl.1824.0016

